# Outcomes of transatrial transcatheter mitral valve replacement with a balloon-expandable valve for severe mitral annular calcification

**DOI:** 10.1016/j.xjtc.2026.102197

**Published:** 2026-01-16

**Authors:** Brett F. Curran, Fernando M. Juarez Casso, Hartzell V. Schaff, Nishant Saran, Phillip G. Rowse, Gabor Bagameri, Kevin L. Greason, Richard C. Daly, Mayra Guerrero, Juan A. Crestanello

**Affiliations:** a Department of Cardiovascular Surgery, Mayo Clinic, Rochester, Minn; b Department of Cardiovascular Medicine, Mayo Clinic, Rochester, Minn

**Keywords:** mitral valve, mitral annular calcification, balloon-expandable valve, transcatheter mitral valve replacement, transatrial transcatheter mitral valve replacement

## Abstract

**Objective:**

To evaluate operative and midterm outcomes, including 1-, 3-, and 5-year survival, of transatrial transcatheter mitral valve replacement (TA-TMVR) with a balloon-expandable valve for severe mitral annular calcification (MAC).

**Methods:**

We retrospectively reviewed patients with severe MAC who underwent TA-TMVR from 2014 to 2024 using a balloon-expandable prosthesis.

**Results:**

Twenty-five patients (68% were female, mean age 75 years) had TA-TMVR for mitral valve disease (92% severe stenosis, 52% moderate-to-severe mitral regurgitation). Previous cardiac surgery was common (48%). Median Society of Thoracic Surgeons Predicted Risk of Operative Mortality was 9% (2%-26%). Most patients were New York Heart Association class III or IV (76%). Preoperative left ventricular ejection fraction was 66%. Concomitant procedures were performed in 68% of cases (aortic valve replacement in 11, septal myectomy in 6, other procedures in 9). A SAPIEN 3 valve was used in 24 patients; most were modified with a felt skirt to improve sealing. Anterior leaflet resection was performed in 24 patients. Operative mortality was 12%. Median length of stay was 14 days. Postoperative left ventricular ejection fraction was 64%, and the mean mitral valve gradient was 5 mm Hg. Paravalvular leak were observed in 6 patients; 3 underwent successful transcatheter closure. One of these patients required a percutaneous valve-in-valve for on-going hemolysis. One-, 3-, and 5-year survival was 68%, 59.5%, and 50.6%, respectively.

**Conclusions:**

TA-TMVR with a balloon-expandable valve is a feasible and durable option for high-risk patients with severe MAC and those requiring concomitant procedures, offering an alternative to conventional surgery in anatomically complex or otherwise-inoperable cases.

## Graphical Abstract

**Figure:**
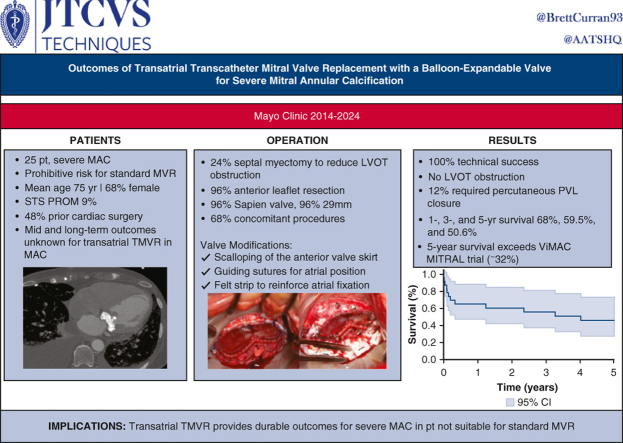

**Figure:**
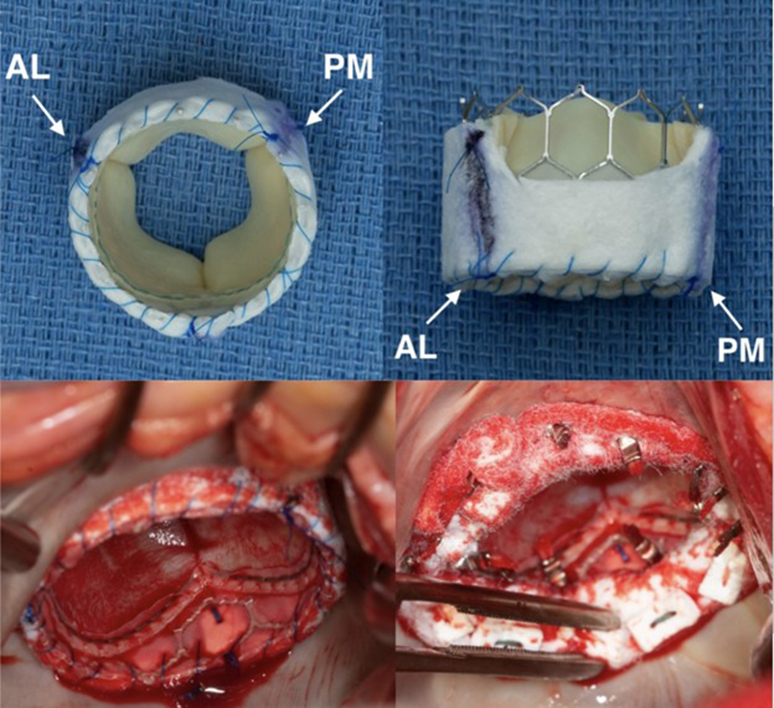
Modified SAPIEN valve for transatrial transcatheter MVR in severe MAC.

**Central Message**

Transatrial transcatheter mitral valve replacement in severe MAC is durable with high technical success and acceptable mortality, offering a viable option for high-risk patients unsuitable for MVR.

**Perspective**

Mitral valve replacement in patients with severe MAC remains a complex challenge because of high surgical risk and anatomical constraints. This study demonstrates that transatrial transcatheter mitral valve replacement is durable and offers high technical success with favorable procedural outcomes, reinforcing its role as a valuable option for select high-risk patients with limited alternatives.

Mitral annular calcification (MAC) is a chronic, noninflammatory, degenerative process involving the fibrous support structure of the mitral valve.^1^ Its prevalence is variable and strongly age-dependent. In a study of all the echocardiograms performed at the Mayo Clinic in 2015, MAC was identified in 23% of patients.^2^ The incidence and severity of MAC increases with advancing age and is associated with traditional cardiovascular risk factors such as atherosclerosis and chronic kidney disease.^3^ Although MAC does not always result in clinically significant mitral valve dysfunction, progressive disease may involve the valvular apparatus, leading to mitral stenosis, regurgitation, or mixed disease. It has been found that significant mitral valve dysfunction is more than twice as prevalent in patients with MAC.^2^ Furthermore, MAC is independently associated with increased risk of mortality, myocardial infarction, stroke, and atrial fibrillation.^3^^,^^4^ The greatest mortality is observed in patients with MAC who also have significant mitral valve disease.^2^

Although surgical and transcatheter treatment improves the outcomes of patients with severe mitral valve disease and MAC compared with the use of medical management,^5^ both are associated with significant technical challenges and imperfect outcomes. Surgery is associated with operative mortality, risk of mitral annular disruption, and circumflex artery injury, whereas transcatheter mitral valve replacement (TMVR) is associated with death, paravalvular leak (PVL), device migration, and left ventricular outflow tract (LVOT) obstruction.^6^

In patients with severe MAC who are not candidates for conventional surgical mitral valve replacement (MVR) or for transcatheter transseptal TMVR, the open transatrial (TA) approach using a balloon-expandable aortic valve represents a promising solution. Previous reports of open TA-TMVR have had variable results. There is a need for more robust, single-institution data to assess the feasibility, safety, and mid- and long-term outcomes of this technique in the setting of severe MAC. We report one of the largest single-institutional experiences with TA-TMVR using a balloon-expandable aortic valve in the setting of severe MAC.

## Methods

We reviewed 25 consecutive patients who underwent open TA-TMVR using a balloon-expandable valve for severe MAC from 2014 to 2024 at the Mayo Clinic. The Mayo Clinic institutional review board approved the study (#24-005982, August 7, 2024), and consent was obtained from all patients to participate in research, including consent for publication. Inclusion criteria included the presence of severe MAC with symptomatic mitral valve disease in patients at high or prohibitive technical risk for conventional MVR surgery. The decision against standard MVR was made by an experienced mitral surgeon on the basis of patient characteristics and the extensiveness of MAC. Patients were also considered for TA-TMVR if they required concomitant procedures. Those who did not require additional procedures were reviewed by interventional cardiologists and were considered unsuitable for TMVR because of anatomy, MAC distribution, or risk of LVOT obstruction. Clinical, echocardiographic, cardiac computed tomography (CT), and operative data were collected from the institution's electronic medical records. Vital status was determined from LexisNexis Accurint, which links information from multiple sources, including the Social Security Death Master File and state death records. The reported death date from any of these sources was used; otherwise, patients were considered alive and were censored 2 weeks before the date their vital status was queried.

The primary end point was technical success, defined as successful implantation of the transcatheter valve without need for conversion to conventional surgery or intraoperative mortality. Secondary end points included mortality, presence of PVL, postoperative complications, and subsequent interventions.

### Surgical Technique

The surgical technique for TA-TMVR with a balloon-expandable valve has been previously described.^7^, ^8^, ^9^ Surgical technique evolved over the study period, and refinements included scalloping of the anterior valve skirt to reduce the risk of LVOT obstruction, use of guiding sutures for precise atrial positioning, and the addition of a felt strip to reinforce atrial fixation. We perform the procedure via median sternotomy with the patient undergoing cardiopulmonary bypass. Left atrial access is achieved either through a left atriotomy or a transseptal approach, depending on the patient's anatomy and the need for concomitant procedures. The mitral valve is assessed, with particular attention to the extent, distribution, and severity of MAC. A CT scan taken preoperatively is reviewed in all cases to assess the predicted neo-LVOT area using virtual valve modeling. A neo-LVOT area <2.0 cm^2^ is considered high risk. Additional high-risk features included basal septal hypertrophy, acute aortomitral angle, and heavy anterior leaflet calcification impeding displacement.^10^

If high risk for LVOT obstruction, a transverse aortotomy is performed, and a transaortic septal myectomy is carried out in a standard fashion. In addition, the anterior mitral leaflet and its chordae is resected to further reduce the risk of LVOT obstruction, whereas the posterior leaflet is left intact.

**Figure 1:**
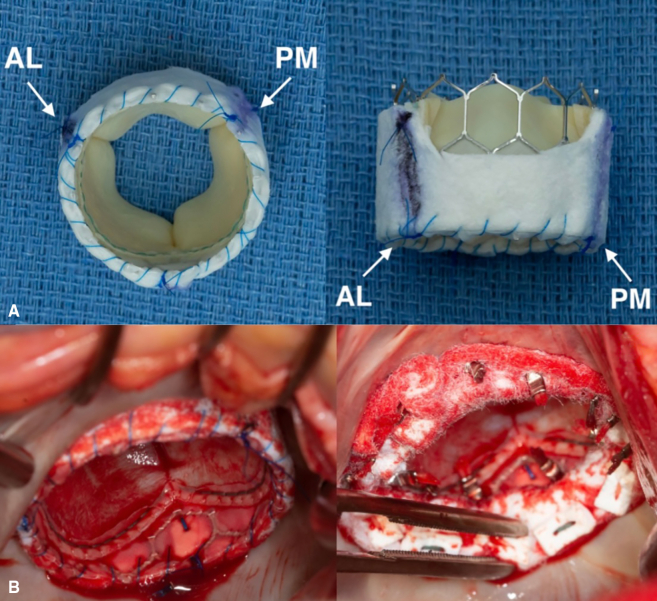
Transatrial transcatheter mitral valve replacement with balloon-expandable aortic valve in a patient with mitral annular calcification. A, Modified SAPIEN 3 valve with a scalloped felt skirt. B, Intraoperative view of implanted and secured prosthesis with and without circumferential pledgeted suture reinforced with felt. *AL*, Anterolateral commissure; *PM*, posteromedial commissure.

The size of the valve is determined by a CT scan taken preoperatively and direct measurement of the mitral annulus diameter. The appropriate-sized SAPIEN aortic valve (Edwards Lifesciences) is selected and prepared on the back table. A felt skirt, approximately 10 to 15 mm in height, depending on valve size, is sewn circumferentially around the inflow portion of the valve using a running 4-0 PROLENE suture (Ethicon) to enhance sealing against the calcified annulus. One third of the skirt is scalloped to avoid LVOT obstruction. The valve is partially crimped with the scalloped portion aligned toward the LVOT (Figure 1, *A*).

Three guiding sutures of 2-0 PROLENE are placed at the anterolateral, posteromedial, and midpoint of the posterior annulus, approximately 120° apart. The guiding sutures are passed through the midsection of the stent at the corresponding commissures of the valve skirt to aid in orientation during deployment and allow for a more atrial position. The valve is then lowered into position and secured with Rummel tourniquets. Care is taken to maintain coaxial alignment during valve descent to prevent tilting or asymmetric deployment, especially in heavily asymmetric MAC.

A balloon is inserted into the SAPIEN valve and inflated to nominal volume to deploy the valve. Balloon inflation is performed slowly under direct visualization. For ease of deployment, we use the Certitude delivery system. After deployment, the 3 guiding sutures are tied. Circumferential 2-0 pledgeted ETHIBOND sutures are then placed through the atrial side of the stent, atrial tissue and an additional felt strip (Figure 1, *B*).

The left ventricle is filled with saline to assess leaflet mobility and detect PVL. Transesophageal echocardiography is performed to confirm valve positioning, assess transvalvular gradients, and verify the absence of PVL or LVOT obstruction.

## Results

### Study Population

**Figure 2:**
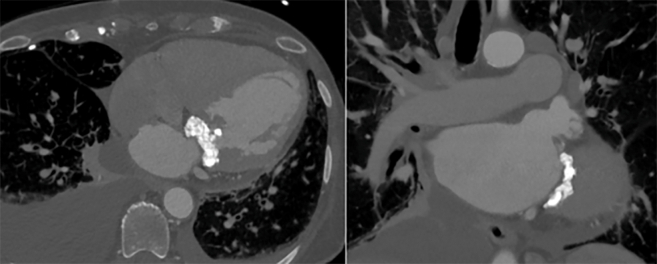
Computed tomography of the chest demonstrating extensive mitral annular calcification.

Among the 25 patients included in the study, 17 (68%) were female, the mean age was 75 years, and all patients had severe MAC (Figure 2). The average MAC score among patients in whom it was calculated was 9.5. Patients were considered prohibitive risk for conventional MVR if annular calcification was extensive and circumferential, such that annular decalcification and reconstruction would carry unacceptable risk of atrioventricular-groove disruption or injury to adjacent structures. Other prohibitive features included deep extension into the subannular left ventricular myocardium, bulky leaflet calcification that limited adequate annular suture placement, and anticipated inability to implant an adequately sized prosthesis without patient-prosthesis mismatch. In all cases, the final decision was made by an experienced mitral surgeon on the basis of annular anatomy and overall operative risk. The average body mass index was 32.3 kg/m^2^. The majority of patients (76%) were New York Heart Association functional class III or IV preoperatively. Twelve patients (48%) had undergone previous cardiac surgery, including coronary artery bypass grafting (n = 4, 16%), aortic valve replacement (AVR; n = 4, 16%), mitral valve repair (n = 4, 16%), and tricuspid valve repair (n = 1, 4%). The mean Society of Thoracic Surgeons (STS) Predicted Risk of Mortality for MVR was 9% (range, 2%-26%). Demographics and baseline comorbidities are further detailed in Table 1.

**Table 1:** Demographics and baseline characteristics

Variable	Values (N = 25)
Demographic data	
Age, y	75 (70-82)
Female	17 (68)
Body mass index, kg/m^2^	32.3 (25.2-36.9)
History of tobacco use	12 (48)
Preoperative conditions	
STS Predicted Risk of Mortality	9 (6.2-9.4)
NYHA III or IV	19 (76)
Previous cardiac surgery	12 (48)
Previous CABG	4 (16)
Previous AVR	4 (16)
Previous MVr	4 (16)
Previous TVr	1 (4)
Previous PCI	6 (24)
Hypertension	22 (88)
Dyslipidemia	21 (84)
Coronary artery disease	17 (68)
Diabetes mellitus	8 (32)
Chronic lung disease	8 (32)
Peripheral vascular disease	6 (24)
Atrial fibrillation or flutter	5 (20)
Previous PPM placement	4 (16)
CHF exacerbation within 2 wk	3 (12)
History of myocardial infarction	3 (12)
Immunosuppression	3 (12)
Renal dysfunction∗	3 (12)
Hemodialysis	1 (4)
Stroke	1 (4)
Infective endocarditis	1 (4)
Preoperative echocardiography	
Ejection fraction, %	67 (65-74)
Mitral valve gradient, mm Hg	9.5 (7.5-10.5)
RVSP, mm Hg	52 (37-62)
Mitral valve regurgitation	
Trivial	3 (12)
Mild	9 (36)
Moderate	7 (28)
Severe	6 (24)
Mitral valve stenosis	23 (92)
Tricuspid valve regurgitation	
Trivial	5 (20)
Mild	11 (44)
Moderate	5 (20)
Severe	2 (8)

Values are presented as n (%) for categorical values or median (interquartile range) for continuous variables. *STS*, Society of Thoracic Surgeons; *NYHA*, New York Heart Association; *CABG*, coronary artery bypass grafting; *AVR*, aortic valve replacement; *MVr*, mitral valve repair; *TVr*, tricuspid valve repair; *PCI*, percutaneous coronary intervention; *PPM*, permanent pacemaker; *CHF*, congestive heart failure; *RVSP*, right ventricular systolic pressure.

∗ Creatinine >2.0 mg/dL or dialysis dependence.

Preoperative transthoracic echocardiography demonstrated a mean left ventricular ejection fraction of 67% (range, 40%-77%). The mean mitral valve gradient was 9.5 mm Hg (range, 5-12 mm Hg). Mitral stenosis was severe in 23 patients (92%), and 13 patients (52%) had moderate or severe mitral regurgitation. Seven patients (28%) had moderate or severe tricuspid valve regurgitation. The mean pulmonary artery systolic pressure was 51.5 mm Hg (range, 30-87 mm Hg). Baseline echocardiographic data are reported in Table 1.

### Intraoperative Characteristics

The mean cardiopulmonary bypass time was 123 minutes (range, 33-223 minutes). Aortic crossclamp was applied in 23 patients (92%) with a mean time of 93.4 minutes (range, 29-141 minutes). Two patients (8%) underwent on-pump beating-heart TA-TMVR without aortic crossclamping or fibrillatory arrest.

Concomitant procedures were performed in 17 patients (68%). The most common was AVR in 11 patients (44%, all with bioprosthesis). Concomitant coronary artery bypass grafting was performed in 3 patients (12%). One patient (4%) underwent tricuspid valve replacement, another patient (4%) underwent tricuspid valve repair, and 4 patients (16%) had atrial septal defects closed. Septal myectomy was performed in 6 patients (24%) to mitigate the risk of LVOT obstruction. Complete anterior leaflet resection was performed in 23 patients (92%), and 1 patient (4%) underwent partial leaflet resection.

Most valves were modified by adding a felt skirt sewn circumferentially around the inflow portion of the prosthesis to enhance sealing. After technique refinement, the anterior one third of the skirt was scalloped to reduce bulk in the LVOT and oriented toward the anterior mitral annulus to mitigate the risk of obstruction. A SAPIEN 3 transcatheter heart valve (Edwards Lifesciences) was implanted in 24 patients (96%), and 1 patient received a SAPIEN XT valve (4%). Valve sizes were 26 mm in 1 patient (4%) and 29 mm in 24 patients (96%). Valve sizing was determined on the basis of preoperative CT annular measurements, intraoperative direct sizing, and surgeon judgment. Our strategy was to implant the largest feasible valve to minimize risk of PVL while avoiding oversizing and potential annular disruption. Intraoperative characteristics are reported in Table 2.

**Table 2:** Intraoperative characteristics

Variable	Values (N = 25)
Mitral valve anterior leaflet resection	24 (96)
Complete resection	23 (92)
Partial resection	1 (4)
SAPIEN valve implantation	25 (100)
SAPIEN 3	24 (96)
SAPIEN XT	1 (4)
Valve size, mm	
29	24 (96)
26	1 (4)
Concomitant procedures	17 (68)
AV replacement	11 (44)
CABG	3 (12)
TV replacement	1 (4)
TV repair	1 (4)
ASD repair	4 (16)
Septal myectomy	6 (24)
Cardiopulmonary bypass time, min	123 (91-152)
Crossclamp used	23 (92)
Crossclamp time, min	93 (65-123)
Total operative time, min	316 (252-395)

Values are presented as n (%) for categorical values or median (interquartile range) for continuous variables. *AV*, Aortic valve; *CABG*, coronary artery bypass grafting; *TV*, tricuspid valve; *ASD*, atrial septal defect.

### Operative Outcomes

There were no intraoperative mortalities or atrioventricular-groove disruption. One patient experienced a perforation of the lateral wall of the left ventricle from the delivery system that was successfully repaired primarily and reinforced with felt. Operative mortality was 12%. All 3 patients died from multiorgan system failure associated with ischemic bowel. The average length of hospital stay was 14 days.

Delayed sternal closure was used in 4 patients because of hemodynamic instability or coagulopathy. Two patients (8%) required reoperation for bleeding. One area of bleeding was identified at the pericardium, and another was at the aortotomy site. No patients experienced postoperative stroke. Other complications included renal failure requiring dialysis in 6 (25%). Prolonged ventilation (>48 hours) was required in 6 patients (24%), and 7 patients (33%) required permanent pacemaker implantation during the index hospitalization.

Postoperative echocardiography revealed a mean left ventricular ejection fraction of 64% and a mean mitral valve gradient of 5 mm Hg. PVL was identified in 6 patients (24%), including 2 with mild and 4 with moderate regurgitation. Three of these patients underwent successful transcatheter PVL closure at 3 weeks, 1 month, and 4 months postoperatively. Two patients required PVL closure because of hemolysis. The other was experiencing worsening dyspnea. One of the patients who underwent PVL closure had ongoing hemolysis and underwent percutaneous transcatheter mitral valve-in-valve with a 26-mm SAPIEN 3 Ultra valve. No patients developed hemodynamically significant LVOT obstruction, valve migration, or embolization. No additional deaths occurred within 30 days after surgery.

**Figure 3:**
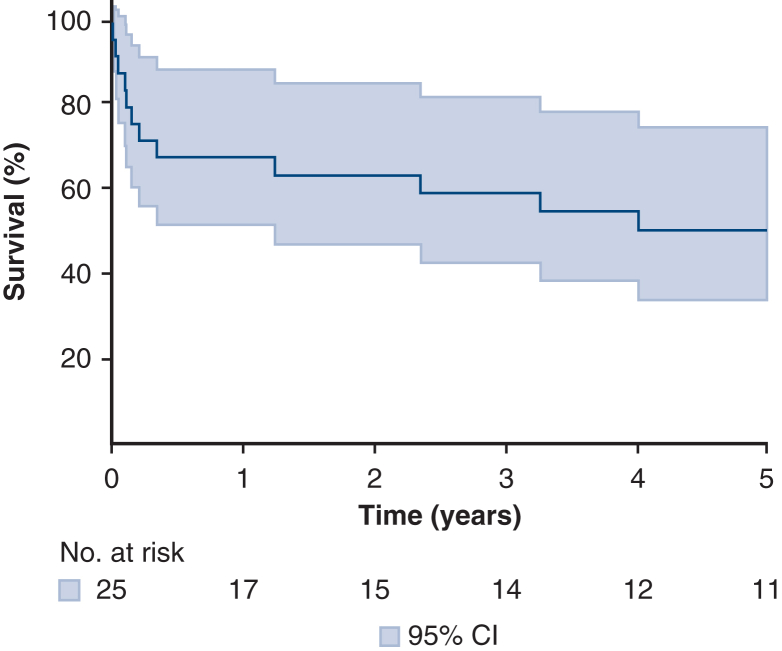
Kaplan-Meier survival curve at 1, 3, and 5 years for patients who underwent transatrial transcatheter mitral valve replacement with 95% confidence intervals in the *shaded areas*.

At 1 year, 8 patients had died, corresponding to a 68% survival rate. This included 3 operative deaths as previously described and 5 additional deaths during follow-up. Of these 5 patients, 2 died from noncardiac death (renal failure and worsening dementia) and 1 died from a cardiac arrest during an endoscopy. The cause of death in the remaining 2 patients was unknown. Survival at 3 and 5 years was 59.5% and 50.6%, respectively (Figure 3). There were no known deaths attributable to structural valve dysfunction, valve thrombosis, endocarditis or other valve-related complications during follow-up. Postoperative outcomes are reported in Table 3.

**Table 3:** Operative outcomes and 1-year outcomes

Variable	Values (N = 25)
Operative mortality	3 (12)
1-year mortality	8 (32)
Length of stay, d	14 (8-16)
Reoperation for bleeding	2 (8)
Delayed sternal closure	4 (16)
Dialysis	6 (24)
Prolonged ventilation (>48 h)	6 (24)
PPM implantation	7 (33)
Paravalvular leak	6 (24)
Mild	2 (8)
Moderate	4 (16)
Paravalvular leak closure	3 (12)
Valve-in-valve	1 (4)

Values are presented as n (%) for categorical values or median (interquartile range) for continuous variables. *PPM*, Permanent pacemaker.

## Discussion

Patients with severe MAC face elevated surgical morbidity and mortality as a result of their underlying disease and associated comorbidities. Although intervention in this population is associated with improved survival compared with medical management, the procedural risks remain substantial, particularly with standard surgical MVR. As a result, operation is often deferred until patients become significantly symptomatic and debilitated. Consequently, the optimal treatment strategy for these high-risk patients remains a clinical challenge for cardiologists and cardiac surgeons.

The least-invasive treatment approach is transseptal TMVR via transfemoral access. Despite its theoretical advantages, the transseptal approach is limited by several procedural challenges, including risk of valve malposition, migration, PVL, and LVOT obstruction. Clinical outcomes are variable, with many studies reporting low technical success and high mortality rates. The Valve-in-MAC Global Registry, 2012-2015, a multicenter study analyzing 116 patients undergoing TMVR via transseptal, transapical, or TA access, found an overall technical success in 76.7% with 14.7% requiring a second valve. LVOT obstruction was seen in 11.2%. The 30-day mortality was 25% and the 1-year mortality was 53.7%.^11^ Among the different approaches, the TA group had a lower risk of mortality at 1 year compared with transseptal and transapical approaches.^11^ This is consistent with the early results of the study, where TA access was noted to have higher technical success and lower 30-day mortality.^12^

A study from the STS/American College of Cardiology Transcatheter Valve Therapy Registry identified 903 patients who underwent valve-in-valve, valve-in-ring, and valve-in-MAC. There were 100 patients who underwent TMVR for valve-in-MAC, of whom only 2% had TA access. LVOT obstruction occurred in 10% of patients, and 30-day mortality was 21.8%. Valve-in-MAC had the lowest procedural success rate and the greatest reintervention rate compared with valve-in-ring and valve-in-valve procedures.^13^ These studies, as well as the study by Yoon and colleagues,^14^ underscore the technical difficulty and elevated mortality risk associated with TMVR in MAC, particularly when performed via transseptal approaches. The authors of the Mitral Implantation of Transcatheter Valve (MITRAL) trial^15^ evaluated all 3 access routes in 31 patients with valve-in-MAC. Sixty-one patients (66%) were excluded because of high risk for LVOT obstruction or valve embolization. Patients were treated with pre-emptive alcohol ablation to help prevent LVOT obstruction, most frequently in patients who underwent a transseptal approach. Among those included, TA technical success was 73.3%, compared with 80% for transseptal access. Thirty-day mortality was 16.7% overall (TA 21.4%, transseptal 6.7%) and 1-year mortality was 34.5% (TA 38.5%, transseptal 26.7%).^15^

Several smaller studies have specifically investigated TA-TMVR. The results are highly variable with differing rates of technical success, complications, and mortality.^8^^,^^9^^,^^16^, ^17^, ^18^, ^19^ Awtry and colleagues^20^ analyzed 222 TA-TMVR cases across 104 hospitals from the STS database from 2014 to 2021. Operative mortality and major adverse cardiac events occurred in 10.4% and 22.5% of patients, respectively.

Despite variability, these dedicated TA studies report consistently high technical success, consistent with our findings. Our study achieved 100% technical success with a 12% 30-day and 32% 1-year mortality. LVOT obstruction was not observed, and 3 patients required percutaneous PVL closure during follow-up. One of those patients required percutaneous valve-in-valve for ongoing hemolysis after PVL closure. Among patients who developed PVL requiring transseptal closure, only trivial PVL was detected intraoperatively on echocardiography, with the greatest degree of periprosthetic regurgitation being mild. PVL occurred more often in the setting of heavy, asymmetric calcification, which limited circumferential sealing despite appropriate valve sizing. The subsequent progression of PVL requiring intervention was most likely related to annular recoil or remodeling. The low incidence of LVOT obstruction in studies on TA supports the benefits of anterior leaflet resection and septal myectomy. The lack of LVOT obstruction is particularly important, because this complication is often fatal, with reported mortality exceeding 50% when it occurs.^11^ Of note, our 3- and 5-year survival rates were 59.5% and 50.6%, respectively. To our knowledge, no previous single-institution series has reported survival at 3 and 5 years after this procedure; most existing publications describe early outcomes only.

Our technique included anterior leaflet resection, septal myectomy when indicated, and anchoring of the valve. We performed skirt modifications to optimize sealing and minimize PVL. We further scalloped the skirt facing the LVOT to reduce its height and mitigate the risk of LVOT obstruction. Guiding sutures were used to achieve optimal valve positioning, allowing deployment in a more atrial position to limit protrusion into the LVOT. In addition, we incorporated a felt strip into the pledgeted sutures, securing the atrial portion of the valve to the surrounding atrial tissue to enhance prosthesis stability and support. These refinements, developed through procedural experience, have contributed to the technical success of the operation. One (4%) of patients with PVL required a valve-in-valve secondary to hemolysis, in contrast to up to 20% in some transseptal series.

Nonetheless, procedural risk remains. We encountered 1 case of left ventricular free wall rupture related to the tip of the delivery system and the usually small left ventricular cavity size in this patient population. This was successfully repaired primarily and reinforced with felt. This underscores the inherent risk of TMVR in MAC, even with TA access. The advanced age and comorbidity burden in our cohort, reflected by high STS Predicted Risk of Mortality, mirrors that of previous studies and contributes to the overall mortality observed. There are indications that outcomes can be improved with better patient selection. This can be seen in the MITRAL trial as 66%, those with unfavorable anatomy, were excluded.^15^ Despite stringent inclusion, at 2 years, approximately 39.3% of valve in patients with MAC in the MITRAL trial died,^21^ and at 5 years the all-cause mortality was found to be 67.9%.^22^ This aligns with the advanced age and comorbidity burden of the MAC population regardless of technique and patient selection. Encouragingly, patients who did have successful intervention were found to have good symptomatic relief up to 2 and 5 years after intervention.^21^^,^^22^

The decision between TA and transseptal TMVR should be individualized on the basis of anatomic considerations as well as patient frailty and comorbidity. Notably, our results and others suggest that when anatomically feasible, the TA approach yields excellent hemodynamic outcomes with fewer device-related complications. Our results highlight the value of the TA approach for patients with unfavorable anatomy for a transseptal approach (hypertrophic basal interventricular septum, narrow neo-LVOT, lack of circumferential calcification to anchor a transcatheter valve) or need for concomitant surgical procedures. Guerrero and colleagues^23^ analyzed MAC scores in 87 patients undergoing percutaneous valve-in-MAC and they found that those with an MAC score ≤6 had a 60% embolization/migration rate, patients with an MAC score ≥7 had a rate of 9.7%, and specifically those with an MAC score of ≥9 had no embolization or migration.

In our series, 11% of patients required concomitant AVR, highlighting an advantage of the open approach in treating complex multivalve disease in 1 operation. Thus, TA-TMVR expands the pool of patients with severe MAC who can be offered definitive treatment of their mitral disease. It is important for surgeons to continue to refine the surgical technique to optimize outcomes. Undoubtedly, the transseptal technique will continue to evolve with adjunctive techniques like LAMPOON (intentional laceration of the anterior mitral valve leaflet to prevent LVOT obstruction), alcohol septal ablation, and dedicated mitral devices.

**Figure 4:**
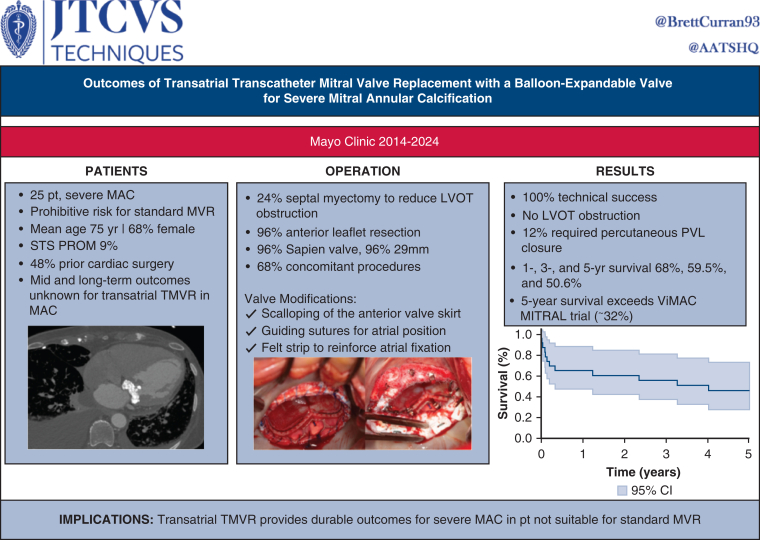
Outcomes of transatrial transcatheter MVR with a balloon-expandable valve for severe MAC. *MVR*, Mitral valve replacement; *MAC*, mitral annular calcification; *STS PROM*, Society of Thoracic Surgeons Predicted Risk of Mortality; *TMVR*, transcatheter mitral valve replacement; *LVOT*, left ventricular outflow tract; *PVL*, paravalvular leak; *ViMAC*, valve-in-mitral annular calcification; *MITRAL*, Mitral Implantation of Transcatheter Valve.

In conclusion, TA-TMVR represents a viable and effective strategy for select patients with severe MAC, particularly those who require concomitant operations. Our findings add to the growing body of evidence supporting this approach, particularly in high-risk patients with complex anatomy (Figure 4). Our results align with the recent American Association for Thoracic Surgery Expert Consensus Document, which emphasizes that patient selection and institutional experience are key determinants of outcomes in mitral surgery for severe MAC. Management of MAC remains technically challenging and carries significant operative risk. The consensus document highlights the complexity of available surgical approaches and underscores the importance of individualized decision-making on the basis of patient risk profile, anatomy, and center expertise. Notably, the document recognizes TA TMVR as a reasonable option in highly selected patients with prohibitive risk for standard MVR. Our study adds novel midterm follow-up data to the limited literature on TA TMVR. To our knowledge, no previous single-institution series has described outcomes beyond the early postoperative period. These findings highlight the potential durability of TA-TMVR in selected high-risk patients and potential advantage over percutaneous TMVR. The 5-year survival in our cohort was 50.6%, which compares favorably with the valve-in-mitral annular calcification (ViMAC) arm of the MITRAL trial, in which 5-year survival was approximately 32.1%. Although differences in patient selection and procedural approach must be acknowledged, these results suggest that TA-TMVR may offer more durable outcomes in appropriately selected patients with severe MAC. The American Association for Thoracic Surgery Expert Consensus Document on mitral valve surgery in MAC underscores the need for additional data on hybrid and transcatheter approaches, and our results provide important evidence to address this gap, particularly as it pertains to late outcomes.^24^ TA-TMVR represents a valuable surgical hybrid approach between conventional open surgery and percutaneous TMVR in anatomically complex patients. Our technique refinements including routine anterior leaflet resection, septal myectomy, valve skirt scalloping, guiding suture positioning, and atrial fixation may serve as a template for centers adopting this approach. With further prospective evaluation and the development of standardized protocols for patient selection and valve preparation, TA-TMVR has the potential to become an integral component of the treatment algorithm for patients with mitral valve dysfunction in the setting of severe MAC.

### Declaration of Generative AI and AI-Assisted Technologies in the Writing Process

During the preparation of this work the author(s) used ChatGPT for grammar correction, scientific writing clarity, and language refinement. The AI tool was not used for data analysis, interpretation, or to generate clinical conclusions. After using this tool/service, the author(s) reviewed and edited the content as needed and take(s) full responsibility for the content of the publication.

## Conflict of Interest Statement

Dr Mayra Guerrero received institutional research grant support from Edwards Lifesciences. All other authors reported no conflicts of interest.

The *Journal* policy requires editors and reviewers to disclose conflicts of interest and to decline handling or reviewing manuscripts for which they may have a conflict of interest. The editors and reviewers of this article have no conflicts of interest.
